# Similarities and differences in gut microbiome composition correlate with dietary patterns of Indian and Chinese adults

**DOI:** 10.1186/s13568-018-0632-1

**Published:** 2018-06-23

**Authors:** Abhishek Jain, Xin Hui Li, Wei Ning Chen

**Affiliations:** 1 Interdisciplinary Graduate School, Nanyang Technological University, 50 Nanyang Avenue, Singapore, 639798 Singapore; 2 Advanced Environmental Biotechnology Centre, Nanyang Environment & Water Research Institute, Nanyang Technological University, 1 CleanTech Loop, Singapore, 637141 Singapore; 3 School of Chemical and Biomedical Engineering, Nanyang Technological University, 62 Nanyang Drive, N1.2-B1-07, Singapore, 637459 Singapore; 4 Zhong Feng International, Hengyang, China

**Keywords:** Next-generation sequencing, Gut microbiota, Prevotella, Bacteroidetes, Asian, Diet

## Abstract

The interaction between diet and gut microbiota, and ultimately their link to health, has turned into the concentration of huge research. However, this relationship still needs to be fully characterized, particularly in case of the Asian population. We compared the fecal bacterial diversity and composition of healthy Indian and Chinese adults, ages 22–35 years, using next-generation sequencing analysis on IlluminaHiSeq 2500 platform. Our analysis revealed unique community structure, dominant *Firmicutes*, *Actinobacteria* and underrepresented *Bacteroides*, of Indian and Chinese gut bacteria. This community structure closely matched with the gut bacterial composition of the Russian population. Therefore, we hypothesized that enrichment of these bacterial clades is supported by high consumption of starch-rich diet such as rice, potato, refined grains. The dominance of genus *Bifidobacterium* due to carbohydrate-rich diet is another notable feature of this study. Moreover, Indian gut bacteria are significantly represented by *Bacteroidetes* (*p *= 0.001) and *Prevotella* (*p *= 0.002) in contrast to Chinese, which could be associated with whole grains and plant-based vegetarian diet of Indians. The gut bacterial population of Indian adults were as diverse as Chinese adults (*p *> 0.1), but significant difference was noticed in gut bacterial composition and relative abundance between two populations (R = 0.625, *p *< 0.005). Partial least squares discriminant analysis and non-metric multidimensional scaling plots showed dietary habit wise clustering of subjects. Thus, the present work confirms an important role of diet in determining gut bacterial composition. LEfse analysis revealed genera *Prevotella*, *Megasphaera*, *Catenibacterium*, *Lactobacillus*, *Ruminococcus* and species *Prevotella copri*, *Lactobacillus ruminis* as the potential biomarkers of diet.

**Electronic supplementary material:**

The online version of this article (10.1186/s13568-018-0632-1) contains supplementary material, which is available to authorized users.

## Introduction

The human gut is a host of trillions of bacteria. The entire gut microbiota is estimated to contain 150-fold more genes than our host genome (Vacharaksa and Finlay 2010). Tremendous progress has been made in linking human gut microbiota with health and disease (Imhann et al. 2018; Qin et al. 2012; Zhao 2013). Imbalance in normal gut microbiota has been associated with inflammatory and metabolic disorders including inflammatory bowel disease (Frank et al. 2007), irritable bowel syndrome (Jeffery et al. 2012), and obesity (Claesson et al. 2012). Therefore, an understanding of what constitutes a health-promoting or disease-promoting microbial group has turned into the concentration of huge research. Gut microbiota composition varies among individuals within and between communities (Conlon and Bird 2015). Several factors such as diet, geography, host genetics and physiology, and drug usage, influence gut microbial composition (Lozupone et al. 2012; Simon and Gorbach 1984; Yatsunenko et al. 2012) but diet has been considered as the most prominent factor amongst all (Chakraborti 2015; Clarke et al. 2012; Harakeh et al. 2016; Lagier et al. 2012; Zhang et al. 2014). Moreover, diet is simplest to modulate and provides the easiest route for therapeutic intervention (Wu et al. 2011). Recent studies have linked diet and microbiome with health (Cardona et al. 2013; Donovan 2017; Singh et al. 2017). Changes in gut microbiota reported in experimental animals fed a high fat diet that induces obesity (Lecomte et al. 2015; Mullin 2010; Murphy et al. 2010). Moreover, controlled diets consist of non-digestible carbohydrates gave to overweight men actuate remarkable changes in certain dominant species, although the responses vary among subjects (Walker et al. 2011).

Over the last 2 decades, microbiome analysis of fecal samples using culture-independent methods, such as high-throughput DNA sequencing has emerged as a non-invasive tool to study nutrition and health (Khanna and Tosh 2014). The development of tools has enabled researchers to explore the interaction between diet and gut microbiota, but this relationship still needs to be fully characterized, especially in case of the Asian population. Research in this area is still at preliminary stages and most of the studies have been performed on European and American population. The diets of Asians vary markedly within the continent and differ substantially from those of other continents. In particular, two most populated countries India and China have unique diet profile. Moreover, India and China together accounted for 15% of world’s total obese population and placed immediately after the US with 13% obese population (Ng et al. 2014). Therefore, it becomes important to characterize the gut microbiota of people from these two countries to further comprehend the correlation between dietary components and the profile of gut bacteria and, eventually, their link to health and disease such as obesity.

In this study, we aimed to (1) characterize the prevalent bacterial taxa to define the community structure in gut samples from healthy Indian and Chinese adults, (2) compare the bacterial diversity distribution within and between the two groups studied, and (3) discover possible relationship of gut bacteria with diet and identify gut bacterial biomarkers of diet which distinguish Indian and Chinese adults. The gut bacterial profiling of 16 healthy Asian adults, including 11 Indians and 5 Chinese, obtained using next-generation sequencing. We further explored the influence of dietary habits on the composition of gut microbiota.

## Materials and methods

### Recruitment of volunteers

A total of 16 healthy adults, including 11 Indians and 5 Chinese, were recruited for the current study (Additional file 1: Table S1). All the volunteers were university students, ages 22–35, studying in Singapore for past 1–3 years. Healthy individuals without any gastrointestinal disorder and who did not use any antibiotics, laxatives or other drugs known to influence gastrointestinal function in the 3 months before the study, were selected. The written informed consent forms and standard questionnaire were taken from the volunteers. Food Frequency Questionnaire (FFQ) was used to recall food diary (Additional file 2). Ethical approval was granted by Nanyang Technological University—Institutional Review Board, Singapore.

### Sample collection

All participants were asked to refrain from smoking, eating, drinking for at least 1–2 h prior to samples collection. Study participants were provided with two different containers: a sterile pot and a 50 mL sterile centrifuge tube. The volunteers were asked to transfer fresh feces from the sterile pot to the tube immediately after defecation. The samples were anonymized in the same order with the questionnaire (e.g. IN1, IN2…IN11 for Indians and CHI, CH2…CH5 for Chinese). Samples were homogenized, 10 g of feces were taken in 50 ml falcon tube and centrifuged (50,000×*g* at 10 °C for 2 h). The fecal water was removed and samples were stored at − 80 °C freezer prior to DNA extraction.

### DNA extraction

0.25 g of frozen fecal sample was used for total genome DNA extraction. DNA was extracted using Mo Bio Powersoil DNA isolation kit (Qiagen). The concentration of DNA sample was measured using NanoDrop and purity was monitored on 1% agarose gel. The concentration of DNA sample was diluted to 1 ng/µL in sterile water.

For each sample, 16S rRNA genes of V4 region were amplified. The primer set corresponding to primers 515F-806R with the barcode was used for amplification. All PCR reactions were performed with Phusion high fidelity master mix (New England Biolabs). The same volume of loading buffer containing SYBR green was mixed with PCR products. Gel electrophoresis with 2% agarose was performed for detection. Samples with the bright main strip between 400 and 450 bp were selected for facilitating experiments. PCR products were mixed in equidensity ratios and afterward purified with Qiagen gel extraction kit (Qiagen, Germany).

### Library preparation and sequencing

Sequencing and analysis were performed by NovogeneAIT (Singapore). Sequencing libraries were generated using TrueSeq DNA PCR-free sample preparation kit (Illumina, USA) according to manufacturer’s recommendations and index codes were added. The library quality was assessed on the Qubit@ 2.0 Fluorometer (Thermo Scientific) and Agilent Bioanalyzer 2100 system. Then, sequencing was performed on an Illumina HiSeq 2500 platform and 250 bp paired-end reads were generated.

### Data analysis

Samples were assigned paired-end reads and reads were merged using FLASH (Magoč and Salzberg 2011). Quality filtering of raw tags was performed according to QIIME quality-controlled process. UCHIME (Edgar et al. 2011) algorithm was used to detect and remove chimera sequences, finally, effective tags were obtained. The Uparse software was used to analyse sequences. Same OTUs were assigned to sequences with ≥ 97% similarity. The representative sequence for each OTU was screened for further annotation. For each representative sequence, the Greengenes database was used based on RDP classifier algorithm to annotate taxonomic information. PyNAST software (Version 1.2) (Caporaso et al. 2010) was used to execute multiple sequence alignment against the “Core Set” dataset in the Greengenes database. Thus, phylogenetic relationship of different OTUs and the distinction of the prevailing species in various samples (groups), were contemplated. Normalization of OTUs abundance information was conducted using a standard of sequence number corresponding to the sample with the least sequences.

Alpha diversity was analysed using, Observed-species, Chao1, ACE, Goods-coverage, Shannon, and Simpson diversity indices. Beta diversity analysis, based on both weighted and unweighted unifrac distances were performed to evaluate compositional heterogeneity among bacterial communities. Unweighted Pair-group Method with Arithmetic Means (UPGMA) hierarchical clustering was performed to interpret the distance matrix using average linkage. All these analyses were conducted by QIIME software (Version 1.7.0) and R software (Version 2.15.3). Non-metric multidimensional scaling (NMDS) based on Bray–Curtis dissimilarity was applied with Analysis of similarities (ANOSIM) and Multi-response permutation procedure (MRPP) to analyse the difference in microbial composition among subjects and test the significance of the difference (Ramette 2007).

### Statistical analysis

Metastats analysis (Paulson et al. 2011), two tailed t test, Wilcoxon two-sample test and Pearson correlation (Xia et al. 2015) were performed. Metastats identifies the differentially abundant feature in metagenomics dataset via nonparametric t test, Fisher’s exact test and False discovery rate (FDR). LEfSe [linear discriminant analysis (LDA) effect size] (Segata et al. 2011) was applied to gut bacterial profiling data to identify bacterial biomarkers differentiating Indians and Chinese based on their dietary habits. The LDA > 4.0 was set as the threshold for selection of features. MetaboAnalyst 3.0 (Xia et al. 2015) was used to perform partial least square discriminant analysis (PLS-DA) to see the separation between gut bacterial profiles of subjects based on their dietary habits.

## Results

To compare the bacterial composition of GI tracts of healthy Asian adults, we collected fresh fecal samples from 16 adults aged 22–35 years old together with food frequency questionnaire. The diet of Indian cohorts mainly consists of whole wheat, rice, lentils, legumes, green vegetables, fruits and dairy products. Apart from this other whole grains, ghee, white flour, and fast food are also substantially included in the Indian diet. Their Chinese counterpart consumed a diet high in animal fat and protein in addition to carbohydrate and vegetables. Chinese food includes rice, noodles, beans, refined grains, white flour, peanut oil, sea food, fish, a lot of variety of meat and animal fat such as lard.

### Taxonomic differences and similarities between gut microbiota of Indian and Chinese adults

**Fig. 1:**
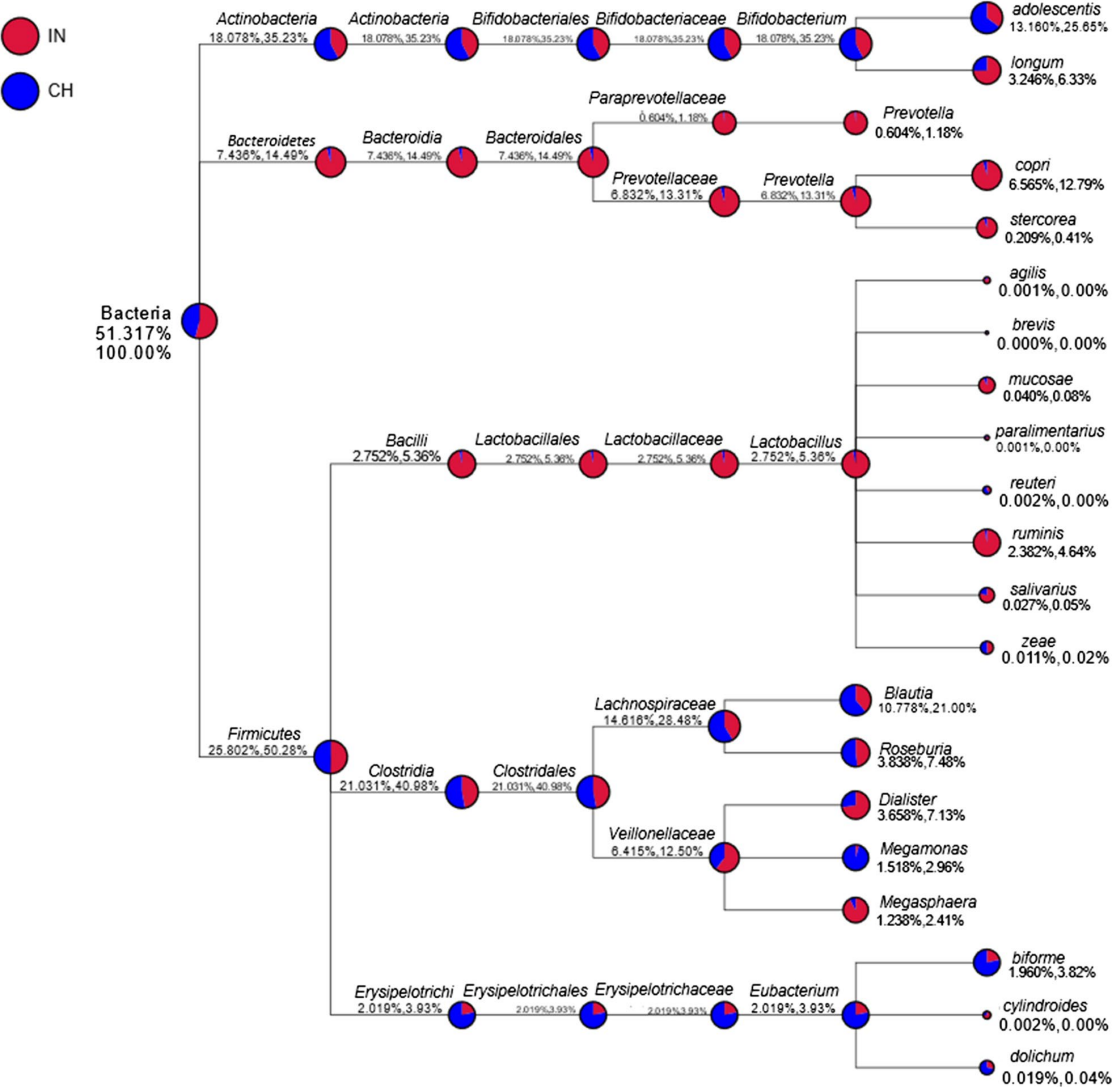
Taxon composition profile of Indian and Chinese gut bacteria. The numbers after the taxonomic ranks are the relative abundances of the corresponding taxon in gut bacteria

Amplicon sequencing on IlluminaHiSeq 2500 platform covering the V4 region of bacterial 16S rRNA of 16 fecal DNA samples representing 11 Indian and 5 Chinese produced on an average of 85,752 high quality reads (average read length 413.12 nt) in each sample (Additional file 3: Table S2). These reads were clustered at 97% similarity threshold into 7223 unique OTUs (388–521 per subject). The classification tree of particularly concerned gut bacteria (top 10 genera for each sample) from both Indian and Chinese samples is shown in Fig. 1. The four major bacterial phyla detected were *Firmicutes*, *Actinobacteria*, *Bacteroidetes and Proteobacteria*, in agreement with previous studies reporting these phyla contributed to the majority of human gut bacteria (Bäckhed et al. 2005; Qin et al. 2010). The dominant taxa at all the taxonomic levels were found to be same in both the groups. The preponderant bacteria were belonging to *Firmicutes* (phylum), *Clostridia* (class), *Clostridiales* (order), *Lachnospiraceae* (family), *Bifidobacterium* (genus) at different taxonomic levels.

**Fig. 2:**
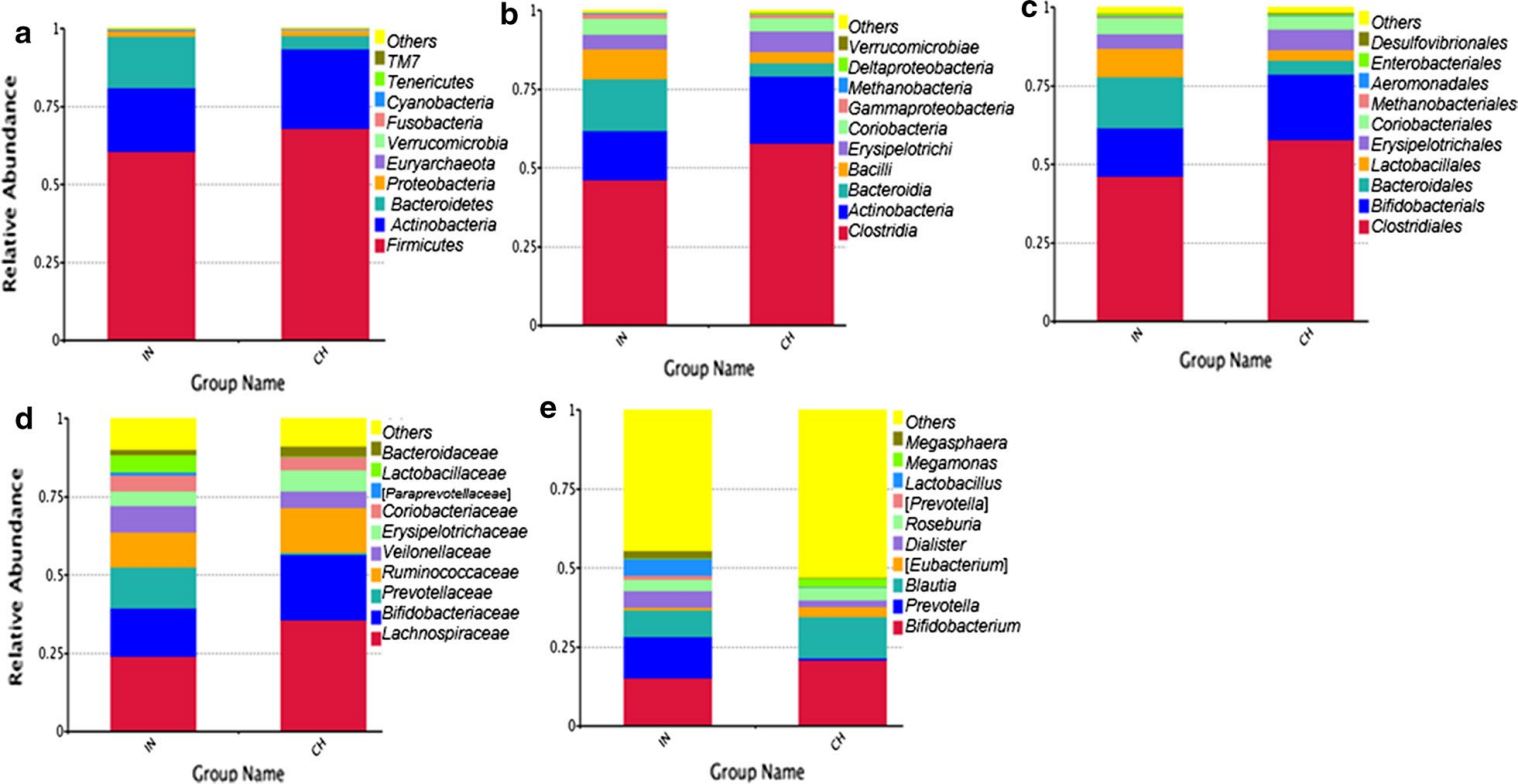
Relative abundance distribution of top 10 bacterial clades from the gut bacterial profiles of Indian and Chinese adults at various taxon levels including **a** phyla, **b** classes, **c** orders, **d** families, **e** genera

The 10 major bacterial clades from the gut bacterial profiles of both the groups at various taxon levels including phylum, class, order, family, genus, and species, are represented in Fig. 2. Metastats analysis and t test were performed to determine species with significant variation between groups (*p *< 0.05) (Additional file 4: Table S3 and Additional file 6: Figure S1). As shown in Additional file 6: Figure S1a, *Bacteroidetes* (p = 0.001) and *Cyanobacteria* (p = 0.003) were significantly higher in Indian group at the phylum level. Particularly, the abundance of *Bacteroidetes* in Indians (16.39%) was almost 4 times higher as compared to *Bacteroidetes in* Chinese (4.27%). The abundance of *Firmicutes* (*68.08*%), *Actinobacteria* (*25.48*%), in Chinese were not significantly different and slightly higher, as compared to *Firmicutes* (*60.5*%), *Actinobacteria* (*20.57*%) in Indians. It is noticeable that *Firmicutes* has outnumbered *Bacteroidetes* in both the populations (Fig. 2a).

The bacterial classes that showed a significant difference (*p *< 0.05) between two groups were *Bacteroidia*, *Bacilli*, *Gammaproteobacteria*, and *Chloroplast*. The bacterial orders (*p *< 0.05) accounting for significant variation between Indians and Chinese were found to be *Bacteroidales*, *Lactobacillales*, *Bacillales*, *Streptophyta*. The bacterial families (*p *< 0.05) *Prevotellaceae*, *Lactobacillaceae*, *Leuconostocaceae*, *Carnobacteriaceae* showed significantly higher abundance in Indians whereas *Porphyromonadaceae* was higher in Chinese.

The bacterial genera (*p *< 0.05) *Prevotella*, *Megasphaera*, *Catenibacterium*, *Lactobacillus*, *Mitsuokella*, *Carnobacterium*, *Lachnospira* were significantly higher in Indians as compared to Chinese. The genera, *Bifidobacterium* (15.2%) and *Prevotella* (13.07%) were almost equally dominant in Indian population (Fig. 2e). *Prevotella* was dominant in 5 Indian subjects whereas rest of the 6 subjects were dominated by *Bifidobacterium*. As shown in Fig. 2e, the two most abundant genera in Chinese were *Bifidobacterium* (20.90%) and *Blautia* (13.19%). Previous studies on different populations have reported that human gut microbiome could be classified based on one of the four distinct communities, namely *Prevotella*, *Bacteroides*, *Clostridiales*, and *Bifidobacterium* (Gorvitovskaia et al. 2016). In our study, both the populations were dominated by *Bifidobacterium* and *Clostridiales* but *Prevotella* in Indians (13.07%) is highly abundant as opposed to very low percentage of *Prevotella* in Chinese (0.58%) (Fig. 2e). Thus, we can conclude that *Prevotella* is a potential biomarker which distinguishes Indian cohorts from Chinese.

**Fig. 3:**
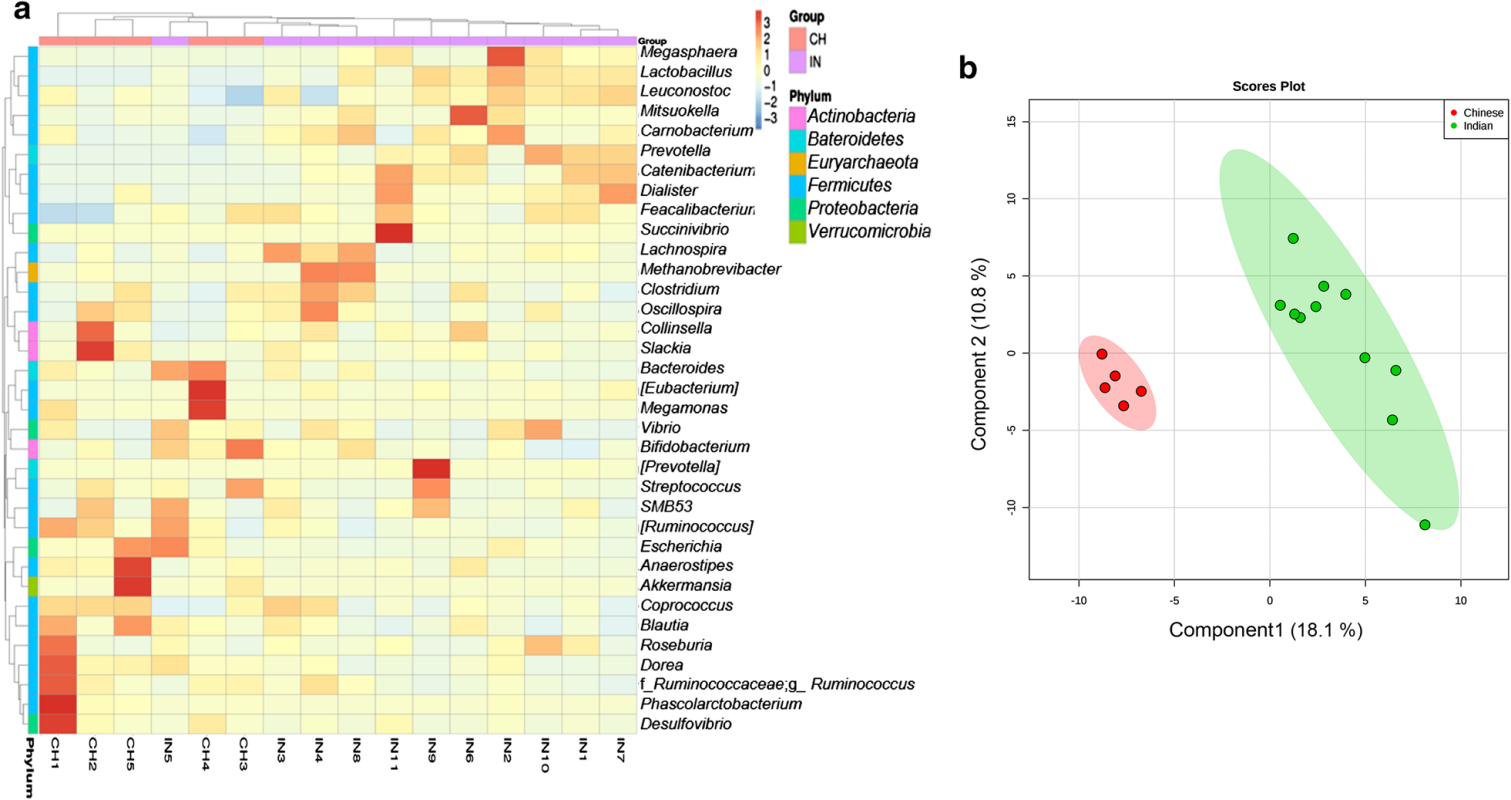
Subjects are clustered based on their dietary habits. **a** Partial least square discriminant analysis of gut bacterial profiles of Indian and Chinese adults. **b** Relative abundance heat map of top 35 genera

To better understand if Chinese and Indians can be separated based on their gut bacterial profiles, hierarchical cluster analysis and partial least squares discriminant analysis (PLS-DA) were performed at all taxa levels (Fig. 3a, b). PLS-DA plot showed clustering of individuals based on dietary habits. Leave-one-out-cross-validation of PLS-DA plot gave R^2^ = 96.56 and Q^2^ = 68.39 which represent variance and predictive capability respectively. We could see differences at all taxonomic levels but the gut bacterial composition at genus level showed the marked difference between two groups. The abundance distribution of dominant 35 genera among all samples was displayed in the heatmap (Fig. 3a). All the Indians clustered together and showed a clear separation from Chinese samples, thus dietary habits played an important role in clustering of the individuals based on the gut bacterial profiles.

### Microbiota from Indian adults is as diverse as microbiota from Chinese adults

**Fig. 4:**
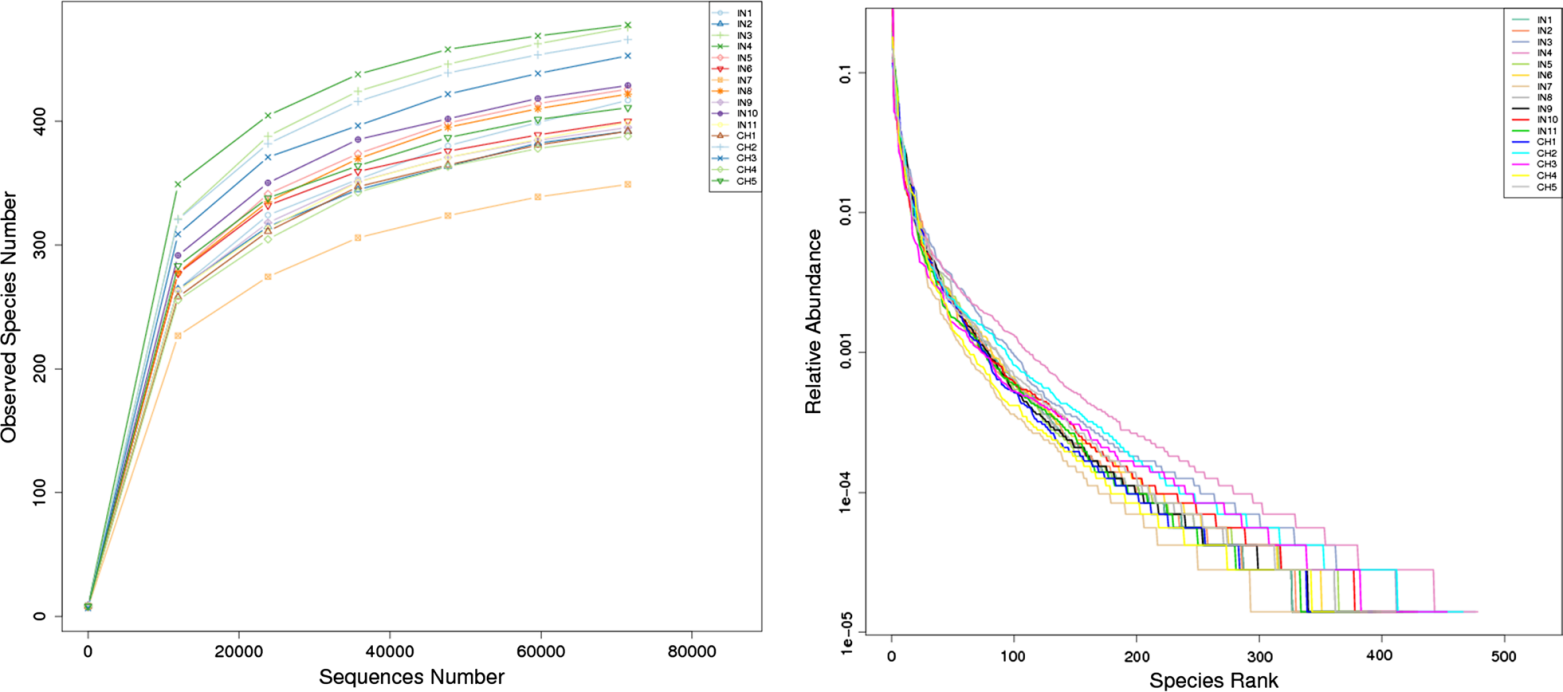
Rarefaction curves and rank abundance curves of alpha diversity: (Left) in rarefaction curves plot, X-axis is number sequencing reads randomly chosen from a certain sample to obtain OTUs. Y-axis is corresponding OTUs. (Right) in rank abundance curves plot, X-axis is the abundance rank. Y-axis is the relative abundance. Curves for different samples are represented by different colours

Alpha diversity is widely used for the analysis of microbial community diversity. It reflects the richness and diversity of microbial community by using a series of statistical indices, species accumulation curve, and species richness curve. As can be seen from Fig. 4, individual rarefaction and rank abundance curves revealed that the Indian adults exhibited similar diversity, including evenness, as the Chinese adults. Alpha diversity between two groups was further compared using observed species, goods coverage, Chao1, ACE, Shannon and Simpson diversity indices. The box plots and tabulated description of statistical indices of alpha diversity are shown in Additional file 5: Table S4 and Additional file 6: Figure S2. Overall, the gut bacterial profiles of Indians and Chinese had the similar value of these statistical indices, as calculated using two-tailed t test with unequal variance and Wilcoxon two-sample test, the *p *> 0.1 for all the indices.

### Gut microbiota composition and relative abundance differ between healthy Indian and Chinese adults

**Fig. 5:**
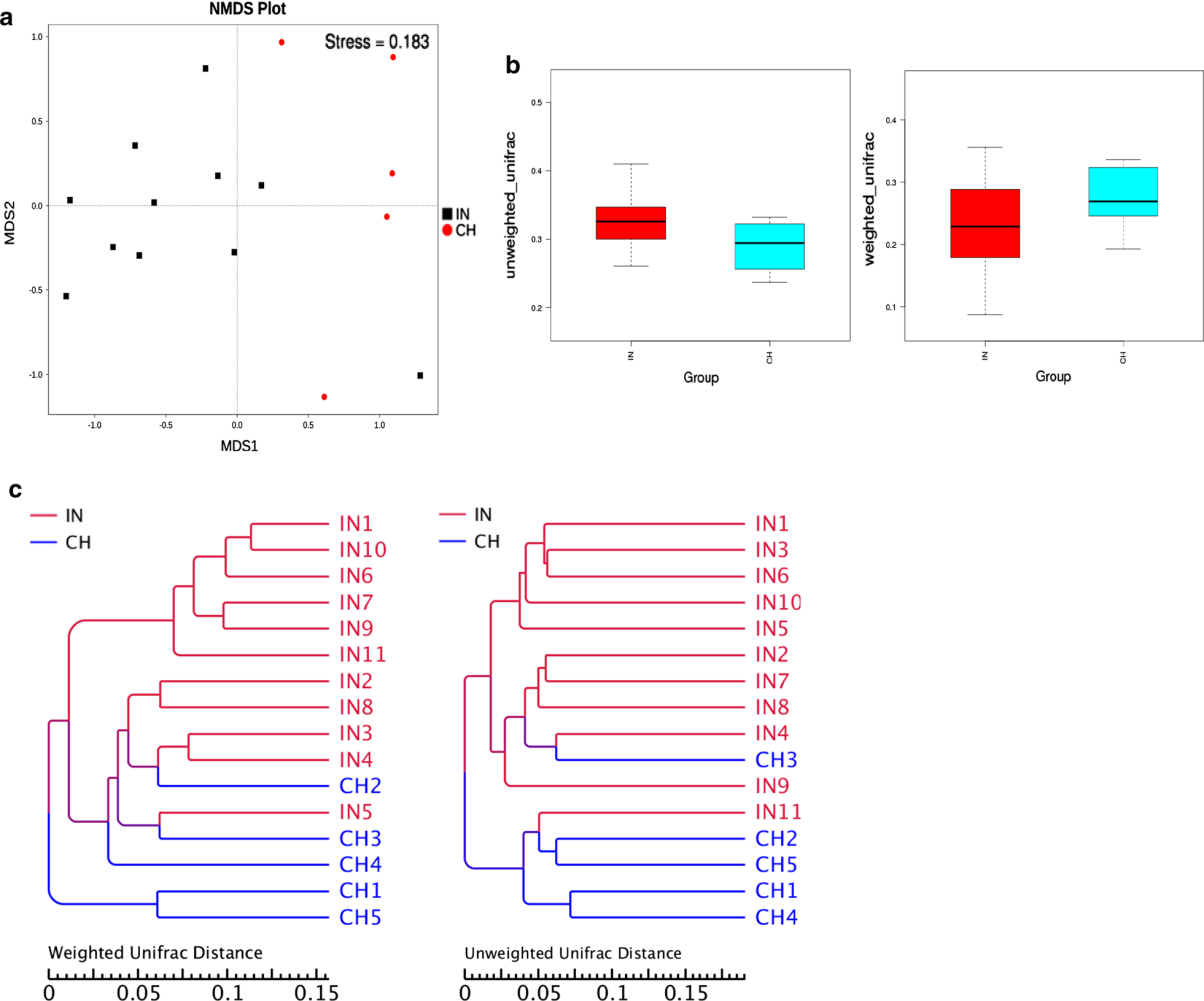
Gut microbiota composition and relative abundance differ between healthy Indian and Chinese adults. **a** NMDS plot of the gut bacterial profiles. Each data point represents a subject. The stress factor value < 0.2 shows the reliability of results. **b** Beta diversity box plots of unweighted and weighted unifrac distances. **c** UPGMA hierarchical clustering based on unweighted and weighted unifrac distances produced distinct clusters which distinguished Indian adults and Chinese adults

The alpha diversity indices were found to be similar in both the groups, now to estimate the extent of variation between gut bacterial profiles of Indians and Chinese, we proceeded to analyse beta diversity using unweighted and weighted unifrac distances. The unweighted unifrac distance is calculated according to phylogenetic relationships of OTUs and weighted unifrac distance is based on the OTU’s abundance information. The beta diversity indices were significantly different (*p *< 0.02) between Indian and Chinese groups, as shown by two-tailed t test and Wilcoxon two-sample test. It reveals that two groups can be distinguished based on both community composition and relative abundance of gut bacteria. The beta diversity indices box plots which describe differences of species diversity between groups are shown in Fig. 5b. Box plots also directly reflect the mean value, the degree of dispersion, the maximum and minimum value which describes intragroup species diversity. Furthermore, the unweighted pair-group method with arithmetic means (UPGMA), using unweighted and weighted unifrac distances, was applied to study the similarities among the subjects. UPGMA hierarchical clustering produced distinct clusters which distinguished Indian adults and Chinese adults (Fig. 5c).

Non-metric multi-dimensional scaling (NMDS) plot of the gut bacterial profiling data indicates the tendency of dietary habit wise grouping of subjects (Fig. 5a). The stress factor value is < 0.2, which confirms the reliability of NMDS analysis results. As shown in Fig. 5a, most of the Indian samples were grouped closely and showed clear separation from Chinese samples, which were more dispersed. The significance of differences was confirmed by the test of analysis of similarity (ANOSIM) (R = 0.620.5, *p *= 0.001), and multi-response permutation procedures (MRPP) (*p *= 0.003). R > 0.5 implies that separation between groups is good and intergroup variation is significantly greater than intragroup variations.

### LEfse analysis identified gut bacterial biomarkers of diet

**Fig. 6:**
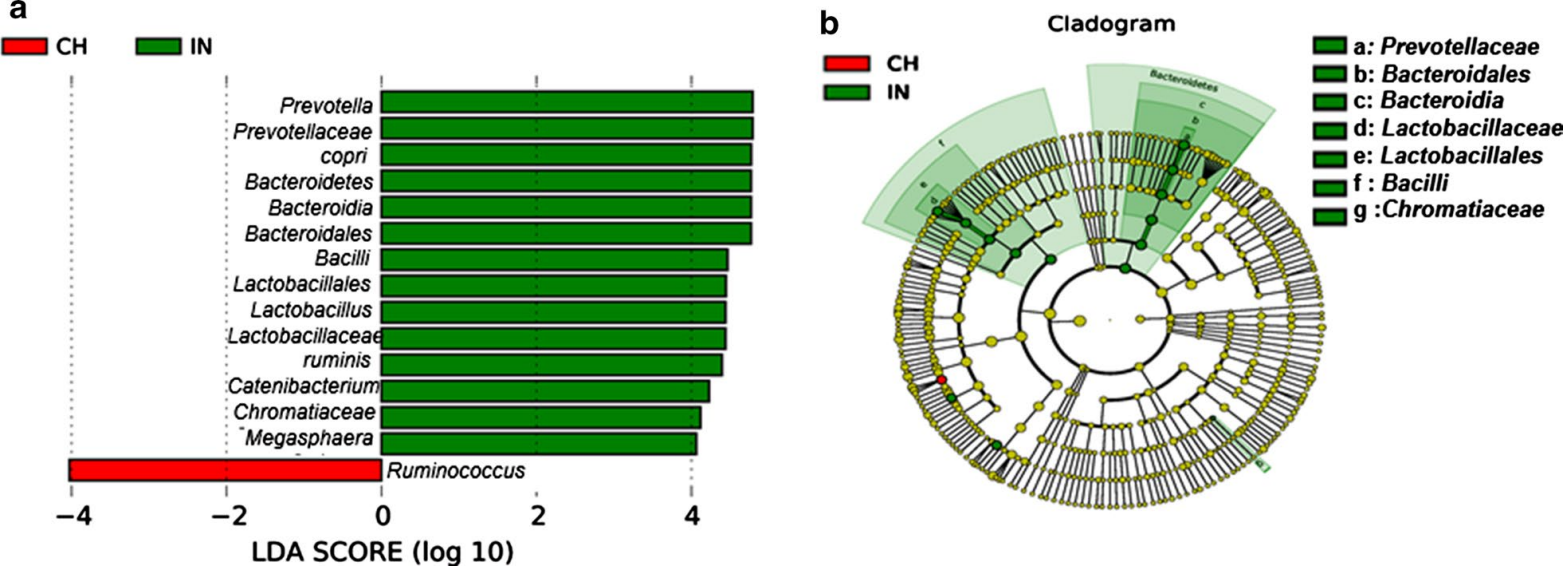
LEfse analysis identified gut bacterial biomarkers of diet. **a** The histogram of the LDA scores presents species (biomarker) whose abundance showed significant differences between Indian and Chinese adults. The length of each bin, namely, the LDA score, represents the effect size i.e. the extent to which a biomarker can explain the differentiating phenotypes between groups. **b** In cladogram, circles radiating from inner side to outer side represents taxonomic level from phylum to genus (species). Each circle’s diameter is proportional to the taxon’s relative abundance. Red nodes refer to the bacteria contributed a lot in Chinese adults, green nodes refer to the bacteria dominant in Indians

Our analysis has revealed a noteworthy contrast in gut bacterial profiles of Indian and Chinese adults. It suggests that different dietary habits could be the major drivers towards distinction in gut microbiota. This led us to examine gut bacterial biomarkers of diet. LEfse [linear discriminant analysis (LDA) effect size] analysis, which couples statistical significance with biological consistency and effect size estimation, detected 15 bacterial clades (LDA score > 4) showing marked differences between Indian and Chinese adults (Fig. 6a). The histogram with LDA scores and cladogram are shown in Fig. 6a, b. In the histogram, green colour represents the taxa found to be more abundant in Indian adults and red colour represents the taxa more abundant in Chinese adults.

The discriminating features at higher taxonomic levels are unlikely to be an effective biomarker, as they incorporate a diversity of genera and species. In this regard, genera *Prevotella*, *Megasphaera*, *Catenibacterium*, *Lactobacillus*, *Ruminococcus and* species *Prevotella copri*, *Lactobacillus ruminis* can potentially serve as biomarkers of diet to distinguish Indian and Chinese adults.

The colours in the cladogram represent the branch of the phylogenetic tree more significantly represents a certain group. In this case, Indians are significantly represented by *Bacteroidetes*.

## Discussion

Long-term diet has been noted as one of the significant factors linked to gut bacterial composition in US and European subjects (De Filippo et al. 2010; Wu et al. 2011). In our study, the unique community structure, dominated by *Firmicutes* and *Actinobacteria* and underrepresented *Bacteroides*, of Indian and Chinese gut bacteria closely matches with the gut bacterial composition of eastern Russia and rural regions consuming starch-rich diet (Tyakht et al. 2013). It is known that a large portion of bacteria belongs to *Firmicutes* and *Actinobacteria*, are nutritionally specialized towards starch (Martens et al. 2011). Presumably, the unique bacterial community is due to high consumption of starch-rich food such as rice, oatmeal or other breakfast cereals, potatoes, refined grains, white flour, noodles, tortillas, beans, in Indian and Chinese adults.

The *Firmicutes* to *Bacteroidetes* (F/B) ratio was 14:1 in Chinese adults and the ratio was 3.7:1 in the Indian adults (Additional file 6: Figure S3). Previous studies have reported that high (F/B) ratio and higher abundance of *Actinobacteria* are associated with obesity (Clarke et al. 2012; Ley et al. 2006; Turnbaugh et al. 2009). The high abundance of *Firmicutes* and *Actinobacteria* (Additional file 6: Figure S4) in Indians (81.07%) and Chinese (93.56%) justifies the study carried out by Ng et al. (2014) in which China and India are placed at second and third position respectively after the US in the world’s most obese population list. Indian and Chinese together accounted for 15% of world’s obese people with 46 million and 30 million obese population respectively.

Another notable feature of our study was the dominance of genus *Bifidobacterium* in gut bacteria of both the communities. *Bifidobacteria* have been reported to confer protection against pathogens and maintain immune system and exertion of nutritional effects to the intestinal cells and the host (Turroni et al. 2014; Ventura et al. 2012). The high abundance of *Bifidobacterium* has previously been noted as a particular feature in Asian children, rural Indian tribes, and rural Russian population (Dehingia et al. 2015; Nakayama et al. 2015; Tyakht et al. 2013). It could be conceived that carbohydrate-based Asian diet drives the colonization of *Bifidobacteria*.

### Dominance of *Bacteroidetes*, *Prevotella*, and *Lactobacillus* in gut bacteria of Indians is consistent with their dietary habits

We found that *Bacteroidetes* and *Prevotella* are significantly dominant in Indians as compared to that of Chinese. These bacterial clades have previously been reported to be more prevalent in the populations consuming vegan or vegetarian diet (De Filippis et al. 2016; De Filippo et al. 2010). *Prevotella* is also known to be associated with indigestible carbohydrate-rich diet, particularly grains such as whole wheat, barley (Kovatcheva-Datchary et al. 2015; Wu et al. 2011). US American and Europe population consuming high animal fat and protein diet had shown a significantly lower abundance of *Bacteroidetes* and *Prevotella*, as compared to Egyptian and rural African population which consumed the vegetarian diet rich in carbohydrate and dietary fibers (De Filippo et al. 2010; Shankar et al. 2017). *Prevotella* are known degraders of xylan and other fibrous polysaccharides (Dodd et al. 2011) which justifies their presence in Indian group where a large fraction of diet is comprised of vegetables and whole grains. It could be inferred that differential abundance of *Bacteroidetes* and *Prevotella* is consistent with the difference between Chinese and Indian diet. *Prevotella* is very well known as an important biomarker of diet (Gorvitovskaia et al. 2016).

In our study, *Lactobacillus* was identified as another important biomarker of diet. Past reports have suggested that dairy associated microbes such as *Lactobacillus* can survive the transit through the digestive system (David et al. 2014). It implies that the significantly higher abundance of *Lactobacillus* in Indians could be due to their high intake of milk products (including cheese or paneer, curd, buttermilk and other dairy products) and fermented products containing *Lactobacillus*.

*Catenibacterium*, *Megasphaera*, and *Mitsuokella* were also found to be enriched in Indian gut. Higher abundance of these three genera was noted in Egyptian children when compared to US children (Shankar et al. 2017). Similarly, *Catenibacterium* and *Mitsuokella* were found only in Bangladeshi children but were absent in the gut of US children (Lin et al. 2013) and they have been previously linked to the abundances of *Prevotella* (Noguera-Julian et al. 2016; Shankar et al. 2017) In present study also, *Prevotella* was found to be in positive correlation with *Catenibacterium*, *Megasphaera*, *Mitsuokella* (r = 0.562–0.90). In comparison to Indians, starch-degrading genera, namely, *Ruminococcus*, and *Blautia*, were more abundant in the stools of Chinese, feasibly due to the high preponderance of starch as a dietary polysaccharide in their diet. It concurs with De Filippis et al. (2016) who previously reported a lower abundance of *Prevotella* and higher abundance of *Ruminococcus* in Omnivores. In addition to this, mucin degrading genus *Akkermansia* which has previously been associated with non-vegetarian (Ruengsomwong et al. 2016) was detected in all Chinese samples whereas only 3 out of 11 Indian samples have shown its presence. *Prevotella* has shown a negative correlation with *Ruminococcus*, *Blautia*, *Dorea*, *Megamonas Bacteroides* (−(0.587–0.797)), while a positive correlation with *Faecalibacterium Dialister*, *Lactobacillus* (0.641–0.775). The correlation of *Prevotella* with these bacteria is in agreement with previously reported results (Dehingia et al. 2015; Nakayama et al. 2015).

Overall this study deepens our understanding of gut microbiota composition of healthy adults from two largest Asian countries, India and China. In spite of the fact that, the prevalent gut bacteria were similar to those found in other populaces, we discovered some exceptional community structure also. This information expands our knowledge of healthy human microbiome ecology and serves as a reference point for future epidemiological investigations and translational applications. Moreover, we examined the correlations among gut bacterial community. We further suggested the possible link between diet and gut bacterial composition. We identified bacterial biomarkers of diet, which are very well known for their interaction with diet, distinguishing Indian and Chinese adults. However other hidden factors such as host physiology and genetics may also interact with their microbiota. Thus, Knowledge from this study could aid future research aim to modulate human gut microbiota, with probiotics or synbiotic dietary supplementations, which may provide new approaches to control the diet-microbiota-human health interactions.

## Additional files

**Additional file 1: Table S1.** Sample information on age, gender and ethnicity.

**Additional file 2.** Food Frequency Questionnaire (FFQ).

**Additional file 3: Table S2.** The summarized information of amplicon sequencing of 16 fecal DNA samples.

**Additional file 4: Table S3.** Metastats analysis showed gut bacterial clades with significant variation between Indian and Chinese adults.

**Additional file 5: Table S4.** Alpha diversity indices.

**Additional file 6: Figure S1.** Between groups T test analysis. **Figure S2.** Between group variation of alpha diversity indices. **Figure S3.** *Firmicutes* to *Bacteroidetes* (F/B) ratio in Indian and Chinese adults. **Figure S4.** Relative abundance of *Firmicutes* and *Actinobacteria*.
