# Supplementary material for: Similarities and differences in gut microbiome composition correlate with dietary patterns of Indian and Chinese adults
Source: AMB Express. 2018 Jun 23;8:104. doi: 10.1186/s13568-018-0632-1 (PMC6015586; doi:10.1186/s13568-018-0632-1)
Supplement: Supplementary file 2 — Additional file 2. Food Frequency Questionnaire (FFQ). [file 13568_2018_632_MOESM2_ESM.pdf]

Food Frequency Questionnaire (FFQ)

Subject no:

Name:

Age:
Marital Status:

Gender: M / F

Race: Chinese / Indian

Contact no/ email address:

Breads and Cereals

Food Item<br>How often do you eat the following:		Portion	Number of times eaten<br>Per day	Per week	Per month	Rarely/Never
**Bread**						
1	White bread	1 slice or 1 piece				
2	Whole meal bread	1 slice or 1 piece				
3	Bread with fruits and nuts	1 slice or 1 piece				
**Bread spreads used**						
4	Butter	1 tsp (D2)				
5	Margarine	1 tsp (D2)				
6	Peanut butter	1 tsp (D2)				
7	Jams/honey	1 tsp (D2)				
8	Kaya	1 tsp (D2)				
9	Lard	1 tsp (D2)				
**Other types of breads**						
10	Roti/Chapati (wheat)<br>Naan<br>Prata (All-purpose flour/Maida)	1 piece				
11	Thosai<br>Idli<br>Vadai	1 piece				
	Others					
12	French toast/roti telur/roti john	1 piece				
13	Bread buns with coconut/curry/meat fillings	1 piece				
	**Cereals**					
14	Plain/flavoured breakfast cereals	4 dsp (D1)				
15	Mixed (with fruit/nuts) breakfast cereals	4 dsp (D1)				
**For participants who consume breakfast cereals (#14, 15):**						
A	You have indicated that you eat breakfast cereals. How often do you have breakfast cereals made from whole-grains?	4 dsp (D1)				
B	Oats/oatmeal (raw)	4 dsp (D1)				

Rice and Porridge

Food Item<br>How often do you eat the following:		Portion	Number of times eaten<br>Per day	Per week	Per month	Rarely/Never
**Bread**						
16	white rice/red rice/brown rice	1B1				
17	Plain porridge	1B1				
**Flavoured rice/porridge**						
18	Fried rice	1B1				
19	Chicken/duck rice/Chicken	1 Portion				

	biryani	
20	Mui fan/curd rice	1 Portion
21	Nasi briyani	1 Portion
22	Nasi lemak	1 Portion
23	Claypot rice	1 Portion
24	Glutinuous rice	1 Portion
25	Flavored porridge (e.g. chicken, pork, duck, fish)	1 Portion

Noodles (rice noodles, wheat noodles, bean noodles, pasta)

Food Item		Portion	Number of times eaten			
How often do you eat the following:			Per day	Per week	Per month	Rarely/Never
**Soup noodles**						
26	Fishball/yong tau foo/wanton/prawn/ beef/chicken/ fish slice	1 Portion				
**For participants who consume Soup noodles(#26):**						
C	You have indicated that you eat noodles in soup. How often do you have soup noodles prepared using brown rice beehoon?	1portion				
**N oodles in gravy**						
**27**	Lor mee/mee rebus	1 Portion				
28	Laksa lemak	1 Portion				
29	Mee siam (with coconut milk)	1 Portion				
**Other Noodles**						
30	Instant noodles	1 Portion				
31	Boiled noodles / spaghetti / pasta (plain)	1 Portion				
32	Boiled noodles / spaghetti / pasta with tomato sauce	1 Portion				
33	Boiled noodles / spaghetti / pasta with cream white sauce	1 Portion				
**For participants who consume boiled spaghetti/pasta (#30-33):**						
D	You have indicated that you eat boiled spaghetti/pasta. How often do you have spaghetti/pasta prepared using wholemeal spaghetti/pasta?	1 portion				

Vegetables and Beancurd

Food Item		Portion	Number of times eaten			
How often do you eat the following:			Per day	Per week	Per month	Rarely/Never
**Pale green leafy vegetables (cabbage, pak choy, lettuce, beansprouts, cauliflower etc)**						
34	Stir fried, plain	½ cup				
35	Stir fried, with meat/ seafood	½ cup				
36	Stir fried in oyster sauce	½ cup				
37	Curry/lemak	½ cup				
38	Raw/steamed/in soup	1 cup				

**Dark green leafy vegetables (spinach, kai lan, chye sim, kangkong, broccoli etc)**		
39	Stir fried, plain	½ cup
40	Stir fried, with meat/ seafood	½ cup
41	Stir fried in oyster sauce	½ cup
42	Stir fried in sambal belacan/dried prawns	½ cup
43	Raw/steamed/in soup	1 cup
**Tomatoes, carrots, red/yellow peppers**		
44	Stir fried, plain	½ cup
45	Stir fried, with meat/ seafood	½ cup
46	Curry/lemak	½ cup
47	Raw/steamed/in soup	1 cup
**Legumes/pulses, e.g. beans, peas**		
48	Stir fried, plain	½ cup
49	Stir fried in oyster sauce	½ cup
50	Stir fried in sambal belacan	½ cup
51	Dried legumes (e.g.dhal, dried beans) in gravy	½ cup
52	Raw/steamed/boiled	½ cup
**Mixed vegetables**		
**53**	Stir fried, plain	½ cup
54	Stir fried, with meat/ seafood	½ cup
55	Stir fried in oyster sauce	½ cup
56	Curry/lemak	½ cup
57	Raw/steamed/in soup/ Chinese rojak	1 cup or 1 serving
**Tofu/beancurd**		
58	Fried	½ square
59	Steamed/ in soups	½ square
**Roots/stems (potatoes, sweet potatoes, corn etc)**		
60	Stir fried	1 cup
61	Steamed	1 cup

Fruits

Food Item		Portion	Number of times eaten			
How often do you eat the following:			Per day	Per week	Per month	Rarely/Never
61	Orange/red/yellow fresh fruits	1 serving				
62	Other fresh fruits	1 serving				
63	Fresh fruit juice	1 cup				
64	Bananas	1 medium				
65	Durians	5 seeds				
66	Canned fruits	½ cup				
67	Mixed fruits (dried)	1 serving				

Poultry

Food Item		Portion	Number of times eaten			
How often do you eat the following:			Per day	Per week	Per month	Rarely/Never
68	Steamed	1 serving				
69	Fried	1 serving				

Meat

Food Item		Portion	Number of times eaten			
How often do you eat the following:			Per day	Per week	Per month	Rarely/Never
**Meat-lean**						
70	Steamed	1 serving				
71	Fried	1 serving				
**Meat-lean and fat**						
72	Steamed	1 serving				
73	Fried	1 serving				
**Meat – preserved/cured**						
74	Sausages	One				
75	Ham	1 slice				
76	Bacon	1 slice				
77	Canned (e.g. luncheon meat, corned beef)	Size of 4 square of chocolate				
78	Liver and other innards	Size of 4 square of chocolate				

Fish/Seafood

Food Item		Portion	Number of times eaten			
How often do you eat the following:			Per day	Per week	Per month	Rarely/Never
**Fish**						
79	Steamed	1 serving				
80	Fried	1 serving				
81	Raw	1 serving				
	**Other sea-food**					
82	Steamed	1 serving				
83	Fried	1 serving				
84	Raw	1 serving				

Eggs

Food Item		Portion	Number of times eaten			
How often do you eat the following:			Per day	Per week	Per month	Rarely/Never
**Whole eggs (including salted and century eggs)**						
85	Boiled/poached/in soup/steamed	1 egg				
86	Fried/scrambled	1 egg				
**Egg whites, only**						
87	Boiled/poached/in soup/steamed	1 egg				
88	Fried/scrambled	1 egg				

Desserts/Local Snacks

Food Item		Portion	Number of times eaten			
How often do you eat the following:			Per day	Per week	Per month	Rarely/Never
89	Fried snacks (e.g. you tiao, goreng pisang, Indian rojak, Samosa, Pakodas)	1 piece				
90	Dim sum – steamed (e.g chee cheong fun, dumplings, rice dumplings)	1 serving				

91	Dim sum – fried/deep fried (e.g. fried carrot cake, wanton, char siew puff)	1 piece				
92	Sweet Indian snacks (e.g. burfi, halwa)	1 piece				

Biscuits, Pastries and Cakes

Food Item		Portion	Number of times eaten			
How often do you eat the following:			Per day	Per week	Per month	Rarely/Never
93	Plain biscuits	2 pieces				
94	Cream filled biscuits/shortbread	2 pieces				
95	Puff/flaky pastries (croissants, baked curry puffs etc)	1 piece				
96	Plain butter cake/fruit cake	1 piece				
97	Sponge cakes	1 piece				
98	Cream cakes	1 piece				

Fast Foods

Food Item		Portion	Number of times eaten			
How often do you eat the following:			Per day	Per week	Per month	Rarely/Never
99	Burgers, with beef or chicken	1 serving				
100	Burgers, fish	1 serving				
101	French fries	1 small serving				
102	Pizza	2 slices				
103	Mashed potato with gravy	1 regular				

Sweetened beverages

Food Item		Portion	Number of times eaten			
How often do you eat the following:			Per day	Per week	Per month	Rarely/Never
104	Sweetened beverages (e.g. soft drinks, packet drinks, yoghurt drinks)	1 G2				

All types of Nuts

Food Item		Portion	Number of times eaten			
How often do you eat the following:			Per day	Per week	Per month	Rarely/Never
105	Raw	½ M1 or 1 small pkt				
106	Dry roasted	½ M1 or 1 small pkt				
107	Fried	½ M1 or 1 small pkt				

Titbits/Snacks

Food Item	Portion	Number of times eaten

How often do you eat the following:			Per day	Per week	Per month	Rarely/Never
108	Fried salty snacks (crisps, prawn crackers, keropok, salted biscuits etc)	1 small packet				
109	Ice cream	1 scoop				
110	Chocolate	4 squares				

Milk & Dairy Products

Food Item		Portion	Number of times eaten			
How often do you eat the following:			Per day	Per week	Per month	Rarely/Never
111	Coffee(with & without milk)	1 M1 –D/2 tsp				
112	Tea(with & without milk)	1 M1 –D/2 tsp				
113	Malt beverages (e.g. hot chocolate, Horlicks®, Milo®, Ovaltine®)	1 M1 –D/2 tsp				
114	Full cream milk* (fresh, UHT, powder)	1 G2*				
115	Low fat milk* (fresh, UHT, powder)	1 G2*				
116	Skimmed milk* (fresh, UHT, powder)	1 G2*				
117	Regular yoghurt	1 G1				
118	Low fat (including frozen yoghurt)	1 G1				
119	Cheese	1 slice/4dsp				
120	Paneer	4 cubes				

Soya Products

Food Item		Portion	Number of times eaten			
How often do you eat the following:			Per day	Per week	Per month	Rarely/Never
121	Soya milk (fresh/packet/can)	1 G2				
122	Soya beancurd (tau huay)	1 B1				

Legend

B1: 1 rice bowl (volume of 300ml)

B2: 1 soup bowl (volume of 700ml)

D1: 1 dessert spoon (10ml)

D2: 1 teaspoon (5ml)

G1: Glass (200ml)

G2: Glass (300ml)

M1: Mug (200ml)
