# Supplementary material for: Similarities and differences in gut microbiome composition correlate with dietary patterns of Indian and Chinese adults
Source: AMB Express. 2018 Jun 23;8:104. doi: 10.1186/s13568-018-0632-1 (PMC6015586; doi:10.1186/s13568-018-0632-1)
Supplement: Supplementary file 6 — Additional file 6: Figure S1. Between groups T test analysis. Figure S2. Between group variation of alpha diversity indices. Figure S3. Firmicutes to Bacteroidetes (F/B) ratio in Indian and Chinese adults. Figure S4. Relative abundance of Firmicutes and Actinobacteria. [file 13568_2018_632_MOESM6_ESM.docx]

**Similarities and differences in gut microbiome composition correlate with dietary patterns of Indian and Chinese adults**

**Authors**

**Abhishek Jain^a,b^,  Xin Hui Li ^d^, Wei Ning Chen^c,*^**

^a^ Interdisciplinary Graduate School, Nanyang Technological University, 50 Nanyang Avenue, Singapore 639798, Singapore

^b^ Advanced Environmental Biotechnology Centre, Nanyang Environment & Water Research Institute, Nanyang Technological University, 1 CleanTech Loop, Singapore 637141, Singapore

^c^ School of Chemical and Biomedical Engineering, Nanyang Technological University, 62 Nanyang Drive, Singapore 637459, Singapore

^d^ Zhong Feng International, Hengyang City, China

^*^Corresponding author

Wei Ning Chen

School of Chemical and Biomedical Engineering, Nanyang Technological University

62 Nanyang Drive, N1.2-B1-07, Singapore 637459

E-mail address: [wnchen@ntu.edu.sg](mailto:wnchen@ntu.edu.sg)

**Figure S1**

**Figure S1 Between groups T-test analysis**

The left panel is the abundance of (a) phyla (b)classes (c)families (d)orders (e)genera (f)species, showing significant difference between group variation. Each bar represents the mean value of the abundance. The right panel is the confidential interval of between group variation. The left-most part of each circle stands for the lower limit of 95% confidential interval, while the right-most part is the upper limit.

 **Figure S2**


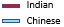


**Figure S2 Between group variation of alpha diversity indices**

(a) ACE (b) Simpson (c) Shannon (d) Chao1 (e) observed Species (f) goods Coverage

**Figure S3**

**Figure S3 *Firmicutes* to *Bacteroidetes* (F/B) ratio in Indian and Chinese adults**

**Figure S4**

**Figure S4 Relative abundance of *Firmicutes* and *Actinobacteria***
